# PRESIDENTIAL SYMPOSIUM: PRESIDENTIAL POLITICS AND THE FORTITUDE FACTOR: EMPOWERING OLDER ADULTS IN TODAY’S POLITICAL LANDSCAPE

**DOI:** 10.1093/geroni/igae098.2091

**Published:** 2024-12-31

**Authors:** Tetyana Shippee, Brian Kaskie

**Affiliations:** Chair; Discussant

## Abstract

In the backdrop of a presidential election, the United States faces a critical issue: a diminishing unity within the advocacy community dedicated to addressing the needs of an aging population. While acknowledging past successes in securing significant public investments for older adults, there is a noticeable decline in advocacy cohesion as the older adults demographic continue to grow. Presenters highlight the need for renewed advocacy efforts, emphasizing intergenerational collaboration, to compel presidential candidates to address aging policy agendas. Presenters also challenge the reliance on individual fortitude in aging policies, arguing that systemic supports are essential to equitable outcomes. They advocate for rethinking aging policies, emphasizing collective action and alternative organizing principles, especially in light of the complex challenges faced by older adults.

